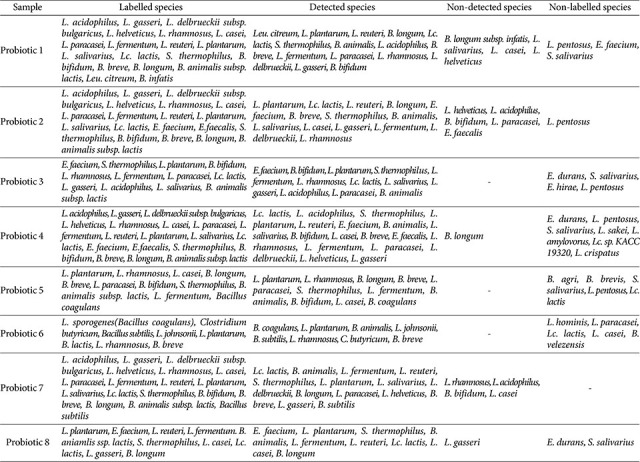# Full-Length 16S rRNA Amplicon Sequencing for the Simple and Simultaneous Detection of Multiple Probiotic Species in Commercial Products

**DOI:** 10.4014/jmb.2603.03005

**Published:** 2026-06-01

**Authors:** Gyeong-Eun Kim, Yoon-Soo Gwak, Chan-Il Bae, Kwangcheol C. Jeong, Mi-Ju Kim

**Affiliations:** 1 Institute of Life Sciences & Resources and Department of Food Science and Biotechnology, Kyung Hee University, Yongin 17104, Republic of Korea; 2 Emerging Pathogens Institute, University of Florida, Gainesville, FL 32611, USA; 3 Department of Animal Sciences, IFAS, University of Florida, Gainesville, FL 32611, USA

**Keywords:** Full-length 16S amplicon sequencing, Probiotic community, Species-level identification, Optimal analysis workflow, Commercial probiotic products

## Abstract

To ensure efficacy and trust, probiotic strains in commercial products should be accurately identified. However, studies have consistently reported discrepancies between labeling and actual microbial composition. Therefore, we developed a full-length 16S rRNA amplicon sequencing workflow for species-level identification of probiotics. To optimize this workflow, four mock communities were analyzed using multiple pipelines and parameters; the workflow accuracy was evaluated. Optimal performance was achieved using Q10 filtering, the Emu pipeline with its default database, and a 0.1% relative abundance threshold. Subsequently, the workflow was applied to eight probiotic products, approximately 50% of which revealed labeling discrepancies with some labeled strains undetected. Furthermore, these results were compared and confirmed using PCR analysis. Despite some discrepancies, both approaches demonstrated overall consistency. Overall, the optimized sequencing workflow facilitates comprehensive species-level identification of probiotics within a single assay, providing a novel approach for quality control and labeling verification.

## Introduction

Probiotics are live microorganisms that confer health benefits on the host when administered in adequate amounts [1]. Commercially available probiotic products worldwide use various probiotic strains. In Korea, the Ministry of Food and Drug Safety (MFDS) has approved 19 probiotic strains, including *Lactobacillus*, *Lactococcus*, *Enterococcus*, *Streptococcus*, and *Bifidobacterium*. Moreover, other strains that have not been approved, including *Pediococcus*, *Weissella*, and *Leuconostoc*, are utilized in probiotic products. Probiotic products contain various strains, and manufacturers provide essential information on the product labels, describing the type of microbes present in the product [2].

As the benefits and safety of microorganisms can vary by strain, accurate probiotic product labeling is crucial for product quality and consumer safety [3]. However, labeling inconsistencies in probiotic products have been repeatedly reported [4, 5]. The absence of listed strains or the presence of unlisted strains comprises these inconsistencies. Such labeling errors diminish the quality and efficacy of the products and raise safety concerns if pathogenic bacteria are included [3]. Consequently, to enhance the quality of probiotic products and provide consumer confidence, the development of effective methodologies for accurate identification and monitoring of microorganisms is essential.

Several studies have been conducted to verify probiotic information accuracy. Most of these studies mainly relied on conventional methods, including PCR and selective culture techniques. However, these methods are time-consuming and limited because they solely depend on the information of specific bacteria listed on the label, making them restrictive in application and unable to provide detailed analyses of the products [2, 4]. Next-generation sequencing (NGS) has recently become an innovative approach for microbial community analysis, overcoming the limitations of conventional methods. The NGS technique enables culture-independent analysis and can accurately identify multiple targets simultaneously through large-scale DNA sequencing [6].

In the analysis of microbial communities using NGS, 16S rRNA amplicon sequencing is employed as the conventional approach. The 16S rRNA gene is approximately 1,550 base pairs in length and contains nine variable regions (V1–V9) that demonstrate significant sequence diversity [7]. Therefore, this gene is broadly recognized as a reliable and efficient standard for taxonomic classification [7, 8].

The 16S rRNA amplicon sequencing of probiotic products is frequently performed using a short-read based sequencing, targeting the V3–V4 region of the 16S rRNA gene [9]. This method facilitates the accurate and rapid identification of diverse bacterial taxa contained in products. However, a short-read based sequencing cannot cover the entire 16S rRNA gene; it only reads partial regions of the 16S rRNA, offering accurate probiotic bacteria identification up to the genus level [7, 10].

Third-generation sequencing has overcome these limitations, enabling full-length 16S rRNA gene sequencing using long-read based sequencers, such as Oxford Nanopore and PacBio [7, 11]. In particular, the MinION sequencer from Oxford is a compact user-friendly device with straightforward operation. Furthermore, this approach has enabled more accurate species-level analysis of microbial communities [6, 12, 13]. However, the application of Nanopore technology to probiotic products remains under-explored. In addition, a standardized analytical workflow ensures accurate and reliable taxonomic profiling has not been established.

Thus, we performed full-length 16S rRNA amplicon sequencing using Oxford Nanopore technology to establish an optimal workflow for accurate probiotic analysis. Rather than developing a new analytical method, this study aimed to establish an optimized workflow by integrating commercially available analysis pipelines with multiple analytical variables. To achieve this, we created four in-house mock communities and sequenced them to assess the accuracy of each sequencing and analysis pipeline. Nanopore sequencing targeted the full-length 16S rRNA gene, and the sequencing results were analyzed using three representative pipelines for 16S rRNA amplicon sequencing: Emu [14], Nanoclust [15], and Epi2me (v 5.1.14, Oxford Nanopore Technologies, UK; https://epi2me.nanoporetech.com/). Each pipeline was applied under various quality score conditions and relative abundance (RA) thresholds. The optimized workflow, confirmed through accuracy evaluation, was subsequently applied to the analysis of commercially available probiotic products, confirming product labeling accuracy.

## Materials and Methods

### Sample Preparation

For 16S rRNA amplicon sequencing, four in-house mock communities containing various probiotic species were utilized (Table 1). Overall, 30 probiotic strains used for constructing the mock communities were obtained from the Korean Collection for Type Cultures (KCTC, Republic of Korea), Korean Culture Center of Microorganisms (KCCM, Republic of Korea), and Korean Agricultural Culture Collection (KACC, Republic of Korea). The *Bifidobacterium* strains constituting the mock community were cultured in BL broth (MB Cell, Republic of Korea) at 37°C for 48 h under anaerobic conditions, and the remaining bacterial strains were cultured in MRS broth (KisanBio, Republic of Korea) at 30°C for 48 h under anaerobic conditions.

Mock 1 comprised 19 MFDS-approved strains, mixed in equal proportions. Mock 2 contained the same 19 strains; however, the proportions were varied. Hypothetically, both Mocks 1 and 2 would demonstrate the detection efficiency of approved probiotics and that the RA of strains would be accurately reflected in samples containing them in either equal or varied proportions. Mock 3 encompassed 11 *Lactobacillus* genus strains from MFDS-approved probiotics, with equal proportions. This mock community was constructed for assessing the ability to distinguish closely related species within the same genus. To evaluate the ability to accurately detect a wide range of probiotic strains, Mock 4 comprised a combination of MFDS-approved probiotics and additional non-approved probiotics, also in equal proportions. The mock community, with a known taxonomic composition, was used as a standardized reference material to comparatively evaluate different analytical workflows and identify the optimal workflow [16].

Eight products were collected from local and online markets in Korea, each labeled with their probiotic composition. The type of products was either powder (n = 6) or capsule (n = 2) form, and all products were utilized for DNA extraction.

### DNA Extraction

The cultured reference probiotic cells were centrifuged at 13,000 rpm for 1 min, and only the pellet was collected. Subsequently, the pellet was used for DNA extraction using the HiGene™ Genomic DNA Prep Kit (BIOFACT, Republic of Korea), with minor adjustments to the manufacturer’s instructions. Furthermore, 100 mg of the probiotic products in powder and capsule forms were resuspended in 1-mL PBS and processed for DNA extraction using similar procedure. A NanoReady Touch spectrophotometer (Life Real, China) was used for measuring the purity of the extracted DNA, and Qubit dsDNA HS Assay kit on the Qubit 4.0 fluorometer (Invitrogen, USA) was used for quantifying the DNA concentration. Moreover, the DNA of the species constituting the mock communities was combined in the proportions indicated in Table 1, and the final concentration of the four mock communities was adjusted to 20 ng/μL.

### Nanopore Sequencing

Full-length 16S rRNA gene amplification using two universal 16S primer sets preceded library preparation for Nanopore sequencing. Universal 16S primer sets were selected to enable detection of all probiotic bacteria present in the community through a single analysis. The primer sets included 27F (AGAGTTTGATCCTGGCTCAG) and 1492R (GGCTACCTTGTTACGACTT) primer set without degenerate bases [17] and 27F (AGRGTTYGATYMTGGCTCAG) and 1492R (CGGYTACCTTGTTACGACT) primer set with degenerate bases [6]. The non-degenerate 27F/1492R primer set has been reported to exhibit reduced amplification efficiency for specific taxa such as *Bifidobacterium*, which may introduce taxonomic bias in microbiome profiling [6]. Therefore, both primer sets were evaluated in this study to ensure a more accurate and representative analysis of community composition. Amplification was performed with the following conditions: 95°C for 3 min, followed by 25 denaturation cycles at 95°C for 30 s, annealing at 55°C for 30 s, extension at 72°C for 2 min, followed by a final elongation cycle at 72°C for 5 min. Subsequently, the PCR products were cleaned using AMPure XP (Beckman Coulter, USA).

The library construction process involved the end prep, native barcode ligation, and adapter ligation steps, using the Native Barcoding Kit 24 V14 (SQK-NBD114.24, Oxford Nanopore Technologies, UK). Using the Qubit dsDNA HS Assay kit on the Qubit 4.0 fluorometer (Invitrogen), the quantity of PCR products and the outcome of each step were measured; the barcoded samples were pooled in equal concentrations. Next, the pooled library was loaded into the R 10.4.1 flow cell (FLO-MIN114, Oxford Nanopore Technologies) and sequenced with MinION (Mk1B). MinKNOW software (v.24.06.16) was operated for sequencing and basecalling using the super-accuracy basecalling model.

### Sequencing Data Analysis

The sequencing reads were filtered using Chopper [18] to filter the read length between 800 to 2,000 bp. Following length filtration, the quality filtering procedure was performed under two conditions as follows: quality scores of Q10 or Q20. Subsequently, the filtered reads were analyzed using pipelines designed for Nanopore 16S amplicon sequencing analysis or those providing dedicated workflows for this purpose: Emu [14], Nanoclust [15], and Epi2me (v 5.1.14, Oxford Nanopore Technologies, UK; https://epi2me.nanoporetech.com/). Emu and Nanoclust were run using default parameters, Emu being executed using either the Emu-developed or SILVA database, and Nanoclust using its default BLAST database. Epi2me was run using two analysis methods within the wf-metagenomic workflow, kraken2 and minimap2, using either the NCBI 16S or SILVA database. Additionally, the results of each analysis method were interpreted with RA thresholds of 0.01% and 0.1%.

### Optimized Sequencing Workflow Determination

To confirm the optimized sequencing workflow, Nanopore sequencing analysis results were evaluated using accuracy evaluation metrics. These metrics were based on the number of true positives (TPs; species correctly identified in the mock), false positives (FPs; species not present in the mock but incorrectly identified), and false negatives (FNs; species not identified), and they include the following three metrics: precision, recall, and F-score. Precision denotes the proportion of claimed TPs that are truly present in the samples, and it is expressed as TP/(TP + FP). Recall refers to the percentage of expected positives that were detected by the workflow, calculated as TP/(TP + FN). F-score is the harmonic mean between the precision and the recall, which can be calculated using the formula 2 × (precision × recall)/(precision + recall). The method demonstrating high values across all three metrics was selected as the optimized sequencing workflow.

### Optimized Sequencing Workflow Application and Validation for Probiotic Product Monitoring

Probiotic products underwent the optimized sequencing workflow, and the results were evaluated for species both labeled and identified, species identified but not labeled, and species labeled but not identified to assess probiotic labeling accuracy.

Furthermore, conventional PCR-based techniques, which are reported for labeling verification, were employed for cross-validation to verify the accuracy of the established workflow in identifying probiotic species. All eight products underwent PCR analyses using species-specific primers targeting the 19 MFDS-approved probiotic strains, based on a previous study [19, 20]. This step sought to determine whether the species listed on the label, as well as those not listed but potentially present, were consistently detected by the sequencing and PCR methods, thereby evaluating the accuracy and reliability of the amplicon sequencing approach.

## Results and Discussion

### Mock Community Analysis

**Fig. 1:**
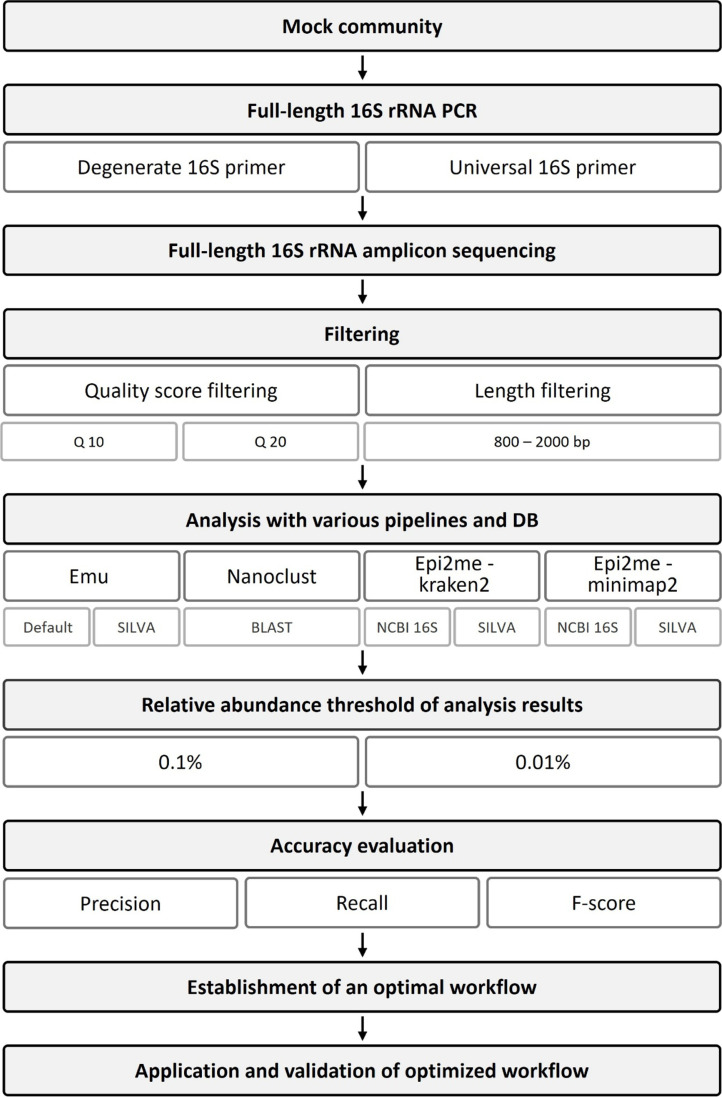
Amplicon sequencing workflow used in this study.

Full-length 16S rRNA amplicon sequencing facilitates species-level analysis of bacterial communities, enabling simple and simultaneous detection of complex microbial structures. Therefore, to develop an optimized sequencing workflow for accurately profiling probiotic bacterial communities, we applied various analysis methods to this sequencing approach, following the workflow detailed in Fig. 1.

Four mock communities with known compositions, each containing various approved and non-approved probiotic strains, were prepared to accurately compare analysis methods and results. Because the taxonomic composition of each mock community was predefined, these communities were suitable for comparing workflow performance under different analytical conditions. Each mock community was amplified with two primer sets and underwent amplicon sequencing. Sequencing data were filtered on the basis of quality score and read length, and the filtered reads were analyzed using Emu, Nanoclust, and Epi2me, providing workflows for Nanopore 16S rRNA amplicon sequencing.

The overall sequencing statistics are presented in Table 2; in all cases except for Mock 1 with universal 16S primer, more than 180,000 reads were obtained following basecalling, demonstrating the high sequencing efficiency of the two primer sets. After filtering the basecalled reads with quality score and length, using Chopper under Q10 and Q20 conditions to retain reads at 800–2,000 bp, the average lengths of the filtered reads were approximately 1,440 and 1,460 bp under Q10 and Q20 conditions, respectively. At this stage, when reads shorter than 800 bp were removed, the number of reads significantly decreased. However, more than 15,000 reads per sample remained after filtering, and these filtered reads were used for subsequent analysis.

**Fig. 2:**
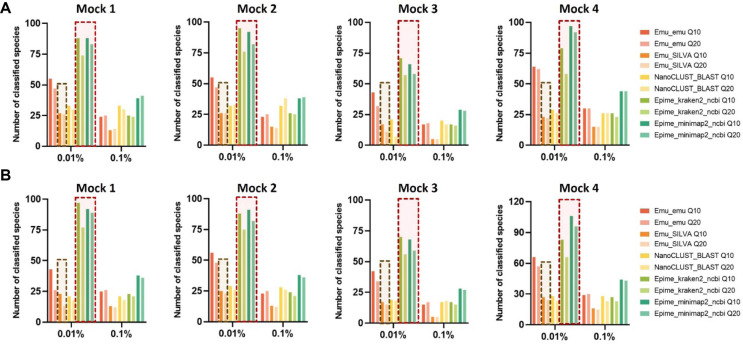
Number of classified species per analysis pipelines using (A) degenerate 16S primer and (B) universal 16S primer.

Subsequently, the differences in species classification among these analysis methods were evaluated by comparing the number of detected species (Fig. 2). The highest number of species was identified with the Epi2me pipeline, which can be attributed to the tendency of Epi2me to classify a greater number of phylogenetically related species, causing the overclassification of species in the mock communities [12]. In contrast, using the SILVA database in Emu, the lowest number of species was detected. This finding may be attributed to the absence of certain species in the SILVA database provided by Emu, limiting the classification of that taxonomy to the genus level. Furthermore, the analysis performed using the SILVA database in Epi2me was performed up to the genus-level classification. This limitation can also be attributed to the identification level provided by the pipeline and database [21].

### Full-Length 16S rRNA Amplicon Sequencing Performance Summary

The performance summary, which lists the TPs, FPs, and FNs for each analysis workflow, was organized for accuracy evaluation. For this evaluation, species-level detection was assumed to be accurate for all included strains, considering that mock communities were fully defined and contained only a single subspecies per species. The summaries in Tables 3 and 4, where degenerate and universal primers are used, respectively, are generally comparable; however, slight differences are noted depending on the analysis condition.

When employing the degenerate 16S primer, most analysis conditions at the species and genus levels demonstrated no FNs, except when Emu was used with the SILVA database. In contrast, using the universal 16S primer caused increased FNs in Mocks 1, 2, and 4. Importantly, the FNs were predominantly concentrated in conditions utilizing the universal 16S primer in the presence of *Bifidobacterium* taxa, indicating primer-dependent detection bias. This observation is consistent with previous reports examining sequence heterogeneities at the non-degenerate 27F primer annealing site of 16S rRNA genes. In those analyses, *Bifidobacterium* showed three base mismatches with the non-degenerate 27F forward primer, whereas most other probiotic taxa did not. Therefore, these mismatches induced biased amplification, consequently resulting in inaccurate detection and underrepresentation of *Bifidobacterium* during microbiome profiling [6].

In addition to primer selection, performance summaries also varied depending on the analytical pipeline and database. When Emu was used with the SILVA database, FN counts were markedly increased at the species level, suggesting a limited coverage of the database. However, all genera were effectively detected under similar analytical conditions, confirming the suitability of this approach for genus-level analysis. When Epi2me was used with the SILVA database, species-level identification was also not achieved, and a greater number of FPs was observed at the genus level than that using the NCBI 16S database with the same analytical method. This is because the SILVA database provided in Epi2me does not exceed the genus level, and therefore the lowest taxonomic rank available for analysis is genus. Furthermore, while the SILVA database is optimized for efficient alignment of short sequences, the NCBI 16S database is highly curated for species-level taxonomy and contains a boarder range of sequences with different lengths and annotation quality [22, 23]. This finding indicates that the choice of taxonomy database can lead to different outcomes at the same taxonomic resolution, despite using the same pipeline.

Along with the pipeline settings, the RA threshold also significantly affected the performance summary. The analysis revealed a substantially reduced number of FPs at the species level when the threshold was set to 0.1%, with Q10 and Q20 conditions generating comparable results. However, Q20 demonstrated a markedly lower number of FPs than Q10 under the 0.01% threshold.

### Accuracy Evaluation and Establishment of the Optimal Analysis Condition

**Fig. 3:**
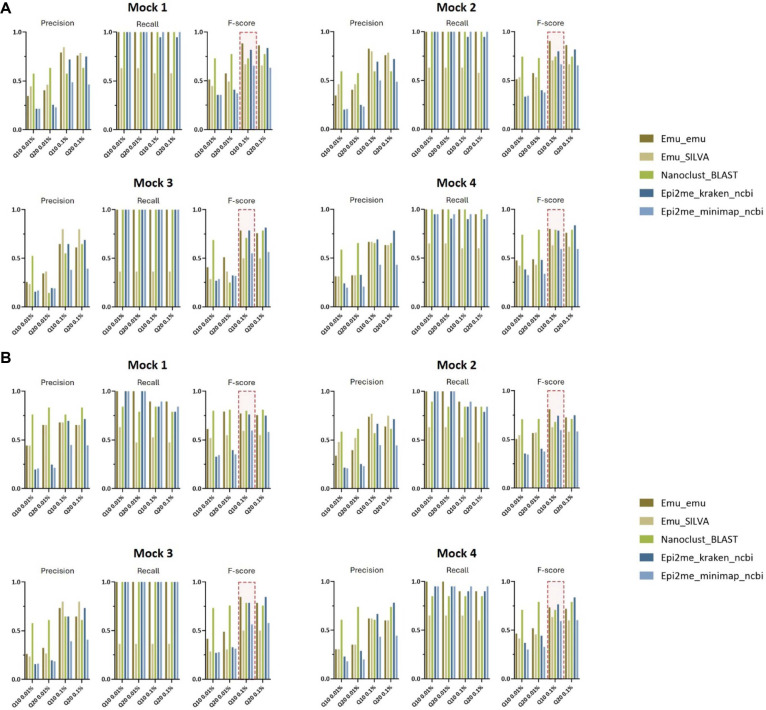
Species-level accuracy evaluation of each pipeline using precision, recall, and F-score with (A) degenerate 16S primer and (B) universal 16S primer.

The accuracy metrics, including precision, recall, and F-score, were calculated on the basis of the performance summary, and the results were used for establishing the optimal analysis condition. The values of the three metrics under different analysis conditions for all mock communities, with higher values indicating greater accuracy, are depicted in Fig. 3. The precision revealed that most pipelines exhibited higher accuracy under the 0.1% RA threshold. Notably, Emu and Epi2me with kraken2 methods displayed particularly high values. Under the 0.01% condition, recall values were slightly higher, with Emu using the SILVA database consistently yielding the lowest recall across all conditions. Moreover, recall values were further reduced when using the universal 16S primer with the 0.1% threshold, which can be attributed to *Bifidobacterium* being classified as FNs. These results demonstrate a clear trade-off between precision and recall depending on the applied RA threshold. Under the 0.1% threshold, the stringent filtering criteria reduced the number of detected taxa, effectively minimizing FPs and enhancing precision. However, this also increased FNs by omitting low-abundance true taxa, thereby lowering recall values. In contrast, the 0.01% threshold improved recall by facilitating the recovery of rare taxa and reducing FNs, while the relaxed threshold also increased FPs and reduced precision. Given the inverse relationship between these two metrics across the RA thresholds, it was difficult to determine the optimal analytical condition using either metric alone. Therefore, the F-score, which represents the harmonic mean of precision and recall and balances the trade-off between the two metrics, was used as the primary criterion for selecting the optimal workflow [24].

Analysis of the F-score revealed that Nanoclust achieved particularly high values under the 0.01% threshold, whereas Emu, Nanoclust, and Epi2me with the kraken2 method exhibited similarly high values under the 0.1% condition. Notably, most workflows yielded higher F-scores at the 0.1% threshold compared with the 0.01% condition. The highest overall F-score was observed under the Q10 and 0.1% RA threshold condition using Emu with its default database and a degenerate 16S primer. Consequently, based on the F-score as a balanced metric for both precision and recall, this analysis workflow was confirmed as the optimal setting and was identified as the optimal sequencing workflow of this study. In addition, this condition was found to be more suitable for species-level verification and quality control of commercial probiotic products, as it effectively reduced FPs of low abundance taxa. The detailed analysis results of the four mock communities, obtained using optimized workflow, are presented in Supplementary Table 1.

### Application of the Established Amplicon Sequencing Workflow

To assess potential labeling discrepancies, the optimized workflow established through mock community analysis was applied to eight probiotic products. Therefore, PCR was performed using degenerate 16S primer, followed by full-length 16S rRNA amplicon sequencing utilizing the Oxford Nanopore platform. Subsequently, the resulting sequencing data were filtered with a quality score of Q10. Taxonomic classification was then conducted using the Emu pipeline with its default database, and the results were filtered at a 0.1% RA threshold. Next, the detected taxa were compared against the species listed on each product’s label. Moreover, in this study, strains specified at the subspecies level, including *Lactobacillus delbrueckii* subsp. *bulgaricus* and *Bifidobacterium animalis* subsp. *lactis*, were identified as successfully detected even when the result resolution was limited to the species level. Based on this comparison, the resulting data were categorized as labeled and detected species (TPs), not labeled but detected species (FPs), and labeled but not detected species (FNs) (Table 5).

Owing to amplicon sequencing, all labeled species were successfully identified in products 3, 5, and 6, indicating the absence of FNs. Conversely, products 1, 2, 4, 7, and 8 showed labeling discrepancies, with between one and five species not detected. These findings suggest that approximately 63% of the tested probiotic products contain labeling inaccuracies, wherein one or more declared species were not detected in the sequencing results. The undetected labeled species showed variation across products, which may be attributed to several factors, including low abundance, PCR amplification bias, or the actual absence of the species. Furthermore, an examination of FPs revealed that, except for product 7, all the other products demonstrated at least one unlabeled species detected with an RA of >0.1%, suggesting contamination or unintended inclusion during production. However, mock community analysis consistently noted FP occurrence, suggesting that such results cannot solely reflect the true composition of the products. This finding may result from sequencing errors, taxonomic misassignments by the analysis pipelines, or technical artifacts, including index swapping during sequencing [13, 25]. Therefore, to determine that the FPs form amplicon sequencing truly reflect components of the products, additional validation is warranted.

### Verification of the Established Amplicon Sequencing Workflow

**Fig. 4:**
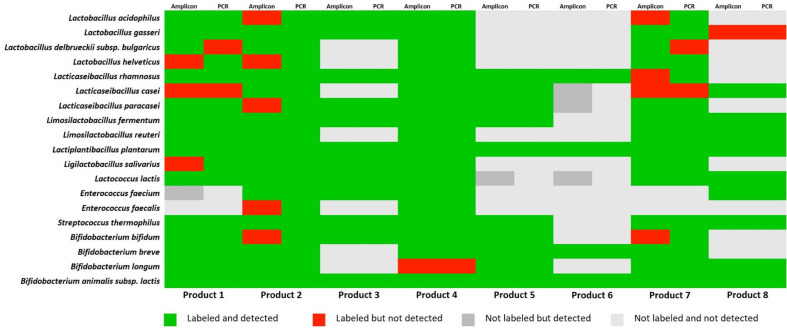
Comparison of the results from the optimized amplicon sequencing workflow and PCR analysis of probiotic products.

We performed PCR analyses, a method that is frequently used as a conventional approach for verifying product labeling, to validate the optimized sequencing workflow and evaluate the labeling discrepancies arising from amplicon sequencing. This study focused on 19 MFDS-approved probiotic strains, based on previous studies [19, 20] and applied across all products. The outcomes of the PCR assays were subsequently compared with the amplicon sequencing results (Fig. 4).

The strains initially classified as FPs in the amplicon analysis, including *E. faecium* in product 1, *Lc. lactis* in product 5, and *L. casei*, *L. paracasei*, and *Lc. lactis* in product 6, were not amplified using PCR, indicating that FPs were not present in the products and were consequence of sequencing artifacts rather than actual labeling discrepancies. Several strains listed on the product labeling but identified as FNs using amplicon sequencing, including *L. helveticus* and *L. salivarius* in product 1; *L. acidophilus*, *L. helveticus*, *L. paracasei*, *E. faecalis*, and *B. bifidum* in product 2; and *L. acidophilus*, *L. rhamnosus*, and *B. bifidum* in product 7, were all successfully detected using PCR. Based on these results, FNs were observed in only three products, and product 2 had the highest number of FNs, reflecting uneven strain abundance among complex multi-species formulation. These PCR results highlighted a major limitation of the amplicon sequencing method: the utilization of strict analysis condition and the impact of amplification bias can hinder the detection of strains that are present in small amounts, significantly impacting detection results. In addition to these observations, several strains demonstrated consistent results between amplicon sequencing and PCR, as in the case of *L. casei*, *B. longum*, *L. casei*, and *L. gasseri* in products 1, 4, 7, and 8, respectively, which were listed but not detected using either method. Accordingly, amplicon sequencing revealed both concordance and discordance with conventional PCR-based detection methods.

Furthermore, as summarized in Table 5, products 5, 6, and 7 contain strains not included in the MFDS-approved list. These non-listed strains exhibited RA values of >0.1% and clearly detected through amplicon sequencing. Although these non-approved strains did not undergo PCR verification, we assumed their presence on the basis of the finding that other strains detected with high RA by amplicon sequencing were consistently amplified using PCR. Therefore, approximately 50% of the tested products (products 2, 3, 5, and 6) contained all the labeled strains, whereas the remaining 50% (products 1, 4, 7, and 8) lacked one or more strains.

However, a discrepancy between amplicon sequencing and PCR results was observed in the case of *L. delbrueckii* subsp. *bulgaricus*. This strain was identified at the species level using amplicon sequencing and presumed to represent the labeled *L. delbrueckii* subsp. *bulgaricus* in products 1 and 7, but not amplified using PCR targeting subspecies-specific markers. These results suggest that a different *L. delbrueckii* subspecies can be present in the product, likely reflecting mislabeling, indicating a limitation of 16S rRNA amplicon sequencing in accurately resolving probiotic strains at the subspecies level. This limitation may arise because genetically closely related subspecies often share highly similar 16S rRNA gene sequences, which may prevent full-length 16S rRNA gene sequencing from providing sufficient resolution to distinguish them [13]. Moreover, the reference database utilized in optimized workflow was predominantly annotated at the species level, causing inadequate subspecies-level identification. Therefore, expanding the reference database to incorporate more detailed taxonomic annotations may enhance the accuracy of full-length 16S rRNA amplicon sequencing in future studies.

Based on these taxonomic constraints, the proposed workflow enables species level identification for probiotic product testing, rather than the subspecies level. However, this still represents an improvement over conventional partial 16S rRNA amplicon sequencing, which generally enables only genus-level identification. Therefore, this enhanced resolution makes the workflow suitable for quality control, especially for verifying product labels and detecting contaminants.

Shotgun metagenomic sequencing can be used as a complementary approach to 16S rRNA amplicon sequencing, enabling more comprehensive microbial profiling. It also allows detection of non-bacterial probiotics such as yeast and assessment of functional features such as antimicrobial resistance (AMR) genes. However, full-length 16S rRNA sequencing offers higher sensitivity for detecting bacterial taxa at low abundance that are often missed by WGS and requires lower sequencing costs and computational resources [3]. Consequently, whereas WGS is valuable for in-depth functional assessment, the full-length 16S rRNA amplicon sequencing workflow provides a more practical, efficient, and user-friendly solution for quality control screening in the probiotic industry.

## Conclusion

This study developed an optimized full-length 16S rRNA amplicon sequencing workflow for accurately and efficiently profiling probiotic microbial communities. Despite limitations in detecting low-abundance strains and resolving subspecies owing to taxonomic resolution constraints, the approach significantly decreased analysis time and complexity by facilitating comprehensive community profiling in a single sequencing run. Moreover, the approach offered superior species-level taxonomic resolution, enabling more accurate identification than conventional amplicon sequencing approaches.

Importantly, this study systematically combined multiple analytical components commonly used in 16S rRNA amplicon sequencing. These include widely applied pipelines such as Emu, Nanoclust, and Epi2me, and analytical parameters such as primer selection and analysis conditions. This approach led to the development of a workflow that provides a valuable alternative to existing methods, offering a simple and accurate approach that can be readily implemented using widely available tools. Therefore, this approach holds considerable potential for broader application as an advanced monitoring system, contributing to the safety assurance of probiotic products and the enhancement of quality control in the industry.

## Supplemental Materials

Supplementary data for this paper are available on-line only at http://jmb.or.kr.



## Figures and Tables

**Table 1:** Composition of mock communities.

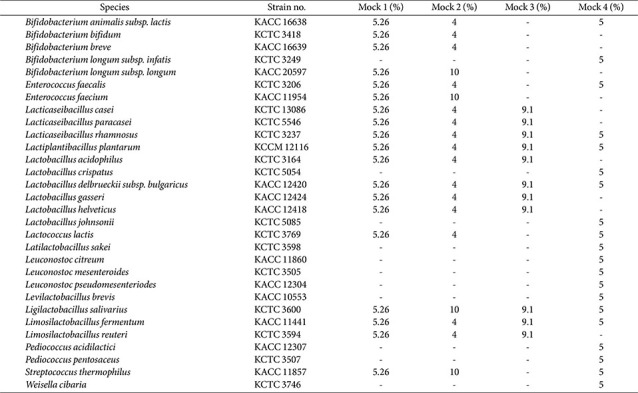

**Table 2:** Statistics of full-length 16S rRNA amplicon sequencing reads.

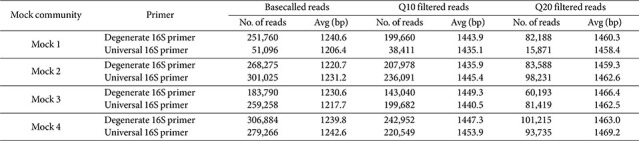

**Table 3:** Performance summary of each pipeline using degenerate 16S primer.

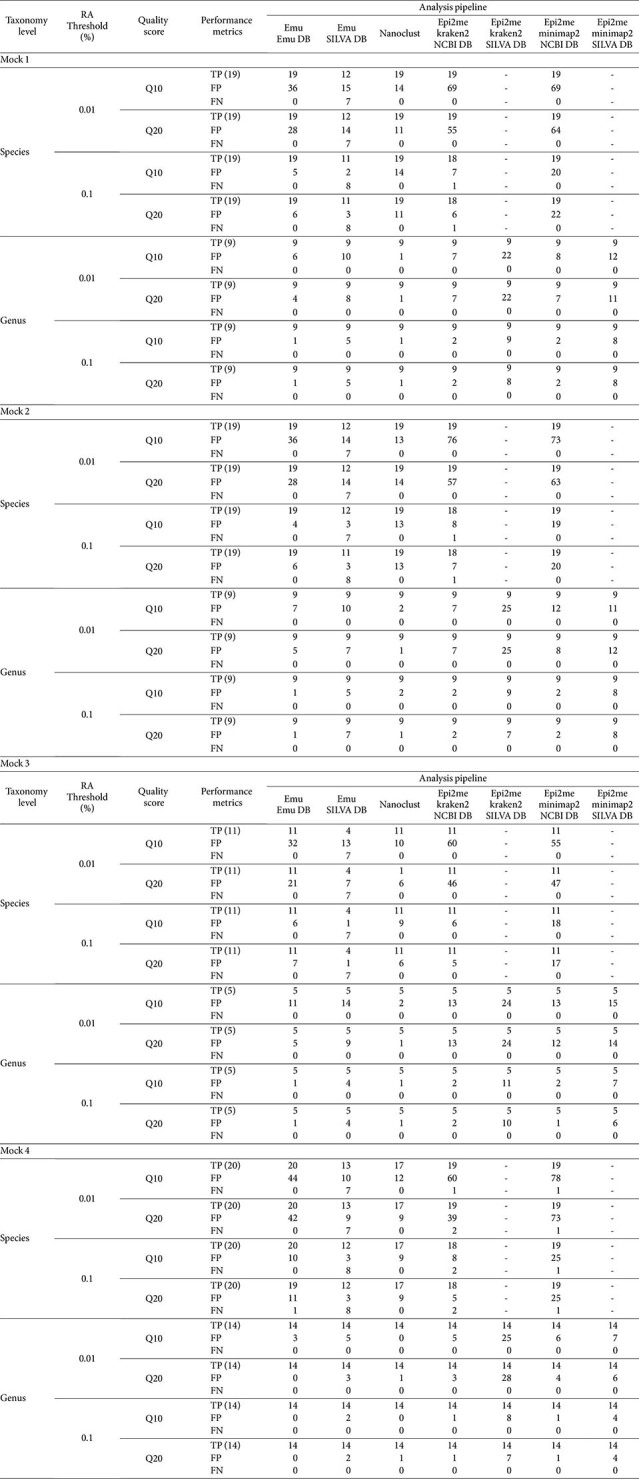

**Table 4:** Performance summary of each pipeline using universal 16S primer.

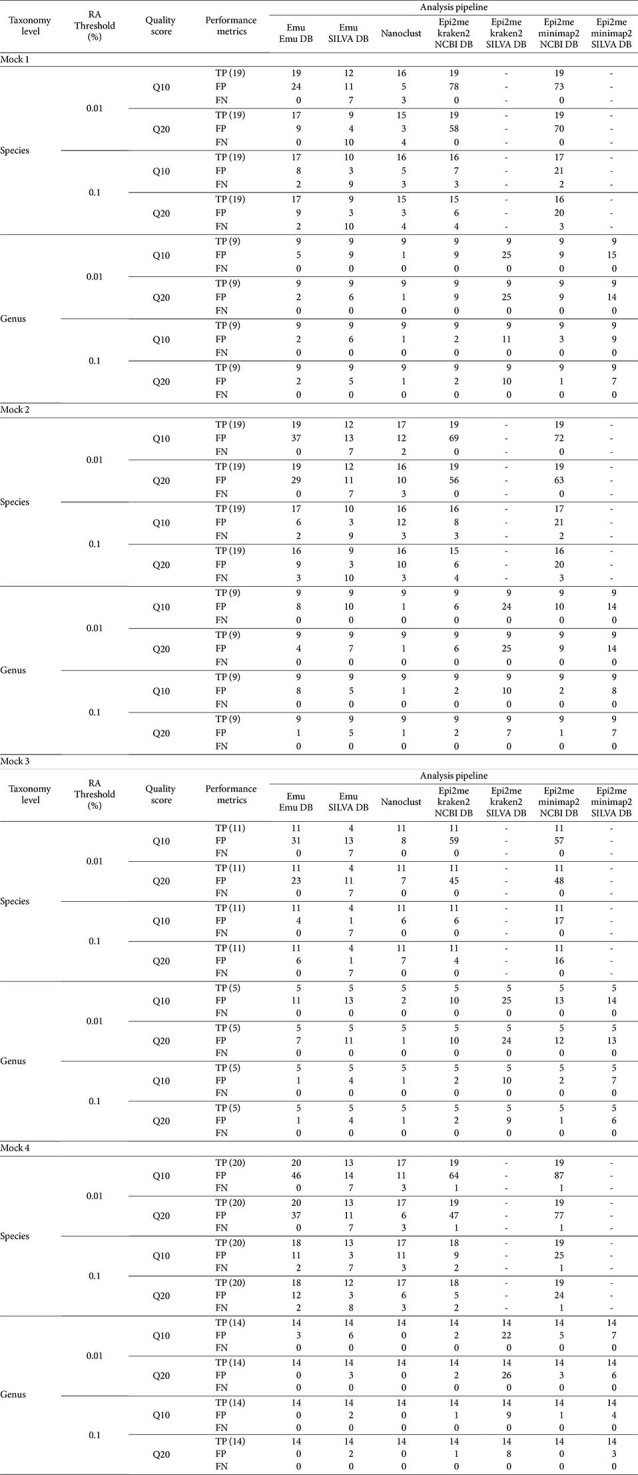

**Table 5:** Application test results of the optimized amplicon sequencing workflow to commercial probiotic products.