# Supplementary material for: Full-Length 16S rRNA Amplicon Sequencing for the Simple and Simultaneous Detection of Multiple Probiotic Species in Commercial Products
Source: J Microbiol Biotechnol. 2026 Jun 1;36:e2603005. doi: 10.4014/jmb.2603.03005 (PMC13246294; doi:10.4014/jmb.2603.03005)
Supplement: Supplementary file 1 [file jmb-36-e2603005-supple.pdf]

J. Microbiol. Biotechnol. 2026. 36: e2603005

https://doi.org/10.4014/jmb.2603.03005

Supplementary Table S1. Relative abundance of mock communities using degenerate 16S primer with Q10 filtered reads at a 0.1% RA threshold in Emu.

Mock 1		Mock 2		Mock 3		Mock 4	
Relative abundance	species	Relative abundance	species	Relative abundance	species	Relative abundance	species
12.62	*Lactobacillus delbrueckii*	11.95	*Lactobacillus delbrueckii*	17.15	*Lactobacillus delbrueckii*	10.87	*Lactobacillus delbrueckii*
11.28	*Lactobacillus gasseri*	9.95	*Lactobacillus gasseri*	14.42	*Limosilactobacillus fermentum*	10.47	*Levilactobacillus brevis*
10.47	*Limosilactobacillus fermentum*	9.54	*Limosilactobacillus fermentum*	14.40	*Lactobacillus gasseri*	9.76	*Limosilactobacillus fermentum*
8.36	*Limosilactobacillus reuteri*	6.60	*Limosilactobacillus reuteri*	11.11	*Limosilactobacillus reuteri*	8.62	*Latilactobacillus curvatus*
7.30	*Bifidobacterium animalis*	6.31	*Bifidobacterium animalis*	8.34	*Lacticaseibacillus rhamnosus*	7.34	*Bifidobacterium animalis*
6.23	*Lacticaseibacillus rhamnosus*	5.81	*Lacticaseibacillus rhamnosus*	7.95	*Lacticaseibacillus paracasei*	5.68	*Pediococcus pentosaceus*
5.84	*Lacticaseibacillus paracasei*	5.46	*Lactococcus lactis*	6.38	*Lactobacillus helveticus*	5.44	*Lactobacillus johnsonii*
5.52	*Lactococcus lactis*	5.31	*Lacticaseibacillus paracasei*	5.26	*Lacticaseibacillus casei*	5.26	*Lactococcus lactis*
4.58	*Enterococcus faecalis*	4.71	*Enterococcus faecalis*	4.87	*Lactiplantibacillus plantarum*	4.70	*Lacticaseibacillus rhamnosus*
4.56	*Lactobacillus helveticus*	4.64	*Ligilactobacillus salivarius*	4.62	*Lactobacillus acidophilus*	4.67	*Enterococcus faecalis*
3.89	*Lacticaseibacillus casei*	4.63	*Lactobacillus helveticus*	3.53	*Ligilactobacillus salivarius*	4.58	*Leuconostoc mesenteroides*
3.31	*Lactiplantibacillus plantarum*	3.54	*Enterococcus faecium*	0.96	*Lactobacillus johnsonii*	3.77	*Pediococcus acidilactici*
2.86	*Lactobacillus acidophilus*	3.53	*Lacticaseibacillus casei*	0.51	*Bacillus velezensis*	3.25	*Leuconostoc citreum*
2.74	*Bifidobacterium breve*	3.49	*Lactobacillus acidophilus*	0.15	*Lactobacillus gallinarum*	2.79	*Ligilactobacillus salivarius*
2.47	*Bifidobacterium bifidum*	3.08	*Lactiplantibacillus plantarum*	0.12	*Lactobacillus crispatus*	2.61	*Weissella cibaria*
2.00	*Enterococcus faecium*	2.83	*Streptococcus thermophilus*	0.11	*Lactobacillus amylolyticus*	2.48	*Lactiplantibacillus plantarum*
1.87	*Ligilactobacillus salivarius*	2.70	*Bifidobacterium bifidum*	0.10	*Bacillus amyloliquefaciens*	2.31	*Lactobacillus crispatus*
1.43	*Streptococcus thermophilus*	2.62	*Bifidobacterium breve*			1.56	*Streptococcus thermophilus*
1.11	*Bifidobacterium longum*	1.88	*Bifidobacterium longum*			1.35	*Leuconostoc pseudomesenteroides*
0.81	*Lactobacillus johnsonii*	0.75	*Lactobacillus johnsonii*			0.60	*Latilactobacillus graminis*
0.37	*Bacillus velezensis*	0.34	*Bacillus velezensis*			0.47	*Lacticaseibacillus paracasei*
0.12	*Streptococcus salivarius*	0.21	*Streptococcus salivarius*			0.29	*Lactobacillus gasseri*
0.11	*Lactobacillus crispatus*	0.12	*Lactobacillus crispatus*			0.23	*Weissella confusa*
0.11	*Lactiplantibacillus pentosus*					0.16	*Lacticaseibacillus casei*
						0.14	*Leuconostoc lactis*
						0.14	*Enterococcus faecium*
						0.14	*Bifidobacterium longum*
						0.11	*Levilactobacillus koreensis*
						0.10	*Latilactobacillus sakei*
						0.10	*Streptococcus salivarius*
